# Examining homoacetogens in feces from adult and juvenile kangaroos with the aim of finding competitive strains to hydrogenotrophic methanogens

**DOI:** 10.1128/spectrum.03183-23

**Published:** 2024-06-21

**Authors:** Renan Stefanini, Supriya Karekar, Fuad Ale Enriquez, Birgitte Ahring

**Affiliations:** 1 Bioproducts, Sciences and Engineering Laboratory, Washington State University, Richland, Washington, USA; 2 Department of Biological Systems Engineering, Washington State University, Pullman, Washington, USA; 3 The Gene and Linda Voiland School of Chemical Engineering and Bioengineering, Washington State University, Pullman, Washington, USA; Temasek Life Sciences Laboratory, Singapore, Singapore

**Keywords:** homoacetogens, methanogens, kangaroo, rumen, *Clostridium*

## Abstract

We examined the microbial populations present in fecal samples of macropods capable of utilizing a mixture of hydrogen and carbon dioxide (70:30) percent. The feces samples were cultured under anaerobic conditions, and production of methane or acetic acids characteristic for methanogenesis and homoacetogenesis was measured. While the feces of adult macropods mainly produced methane from the substrate, the sample from a 2-month-old juvenile kangaroo only produced acetic acid and no methane. The stable highly enriched culture of the joey kangaroo was sequenced to examine the V3 and V4 regions of the 16S rRNA gene. The results showed that over 70% of gene copies belonged to the Clostridia class, with *Paraclostridium* and *Blautia* as the most predominant genera. The culture further showed the presence of *Actinomyces* spp., a genus which has only been identified in the GI tract of macropods in a few studies, and where none, to our knowledge, have been classified as homoacetogenic. The joey kangaroo mixed culture showed a doubling time of 3.54 h and a specific growth rate of 0.199/h, faster than what has been observed for homoacetogenic bacteria in general.

**IMPORTANCE:**

Enteric methane emissions from cattle are a significant contributor to greenhouse gas emissions worldwide. Methane emissions not only contribute to climate change but also represent a loss of energy from the animal's diet. However, methanogens play an important role as hydrogen sink to rumen systems; without it, the performance of hydrolytic organisms diminishes. Therefore, effective strategies of methanogen inhibition would be enhanced in conjunction with the addition of alternative hydrogen sinks to the rumen. The significance of our research is to identify homoacetogens present in the GI tract of kangaroos and to present their performance *in vitro*, demonstrating their capability to serve as alternatives to rumen methanogens.

## INTRODUCTION

Macropods belonging to the family of Macropodidae, mainly kangaroos and wallabies, have been increasingly studied for their microbiota capable of producing acetic acid from hydrogen and carbon dioxide by a process known as reductive homoacetogenesis (1). These microbes are believed to be the reason for the low production of methane in macropods (2). This is in contrast to ruminants such as cows and sheep, where methanogenesis is the prevailing final process producing methane from the foregut anaerobic fermentation (3). With the increasing focus on greenhouse gas emissions, the opportunity for switching the final fermentation of hydrogen and carbon dioxide in the rumen away from the production of methane and preferably into the beneficial production of acetic acid has gained major interest (4). Both macropods and ruminants rely on pre-gastric fermentation for more efficient utilization of the nutrients present in the grass, hay, and other ingested plant materials, while acetic acid and other volatile fatty acids (VFAs) produced in their digestion system are transferred to the bloodstream as their main source of energy for the ruminant animal (5). The microbes in these foregut systems also synthesize essential organic compounds such as vitamins and are a great source of microbial proteins (6). Hydrogen plays a central role in the fermentation happening in their foregut as a principal electron donor, and its accumulation tends to be inhibitory to many upstream hydrolytic processes that break down lignocellulosic material (7).

**Fig 1:**
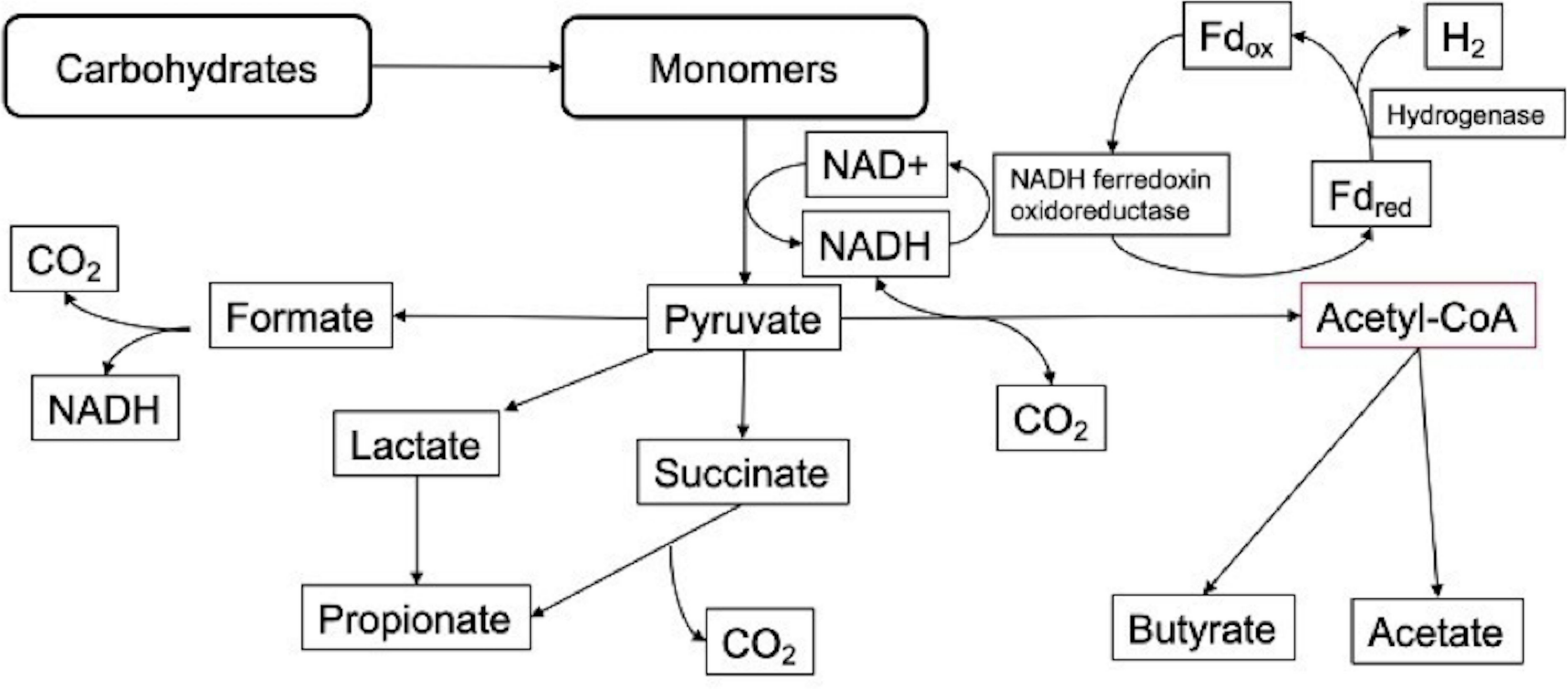
Metabolic pathway of anaerobic carbohydrate fermentation present in the rumen and the main sources of carbon dioxide and hydrogen formation.

Microorganisms that are capable of consuming hydrogen play a crucial role in these systems, where they act as a hydrogen sink, ensuring the stability of the systems, including pH control (8, 9). Research on the microbiota of the macropod’s foregut showed that the populations of methanogens are small, along with limited production of methane gas, while the homoacetogenic populations are dominating. These studies have further been verified by specific studies of metagenomics and isolations showing that most isolates belong to the phylum of Firmicutes (4, 10–12). These bacteria were found to use the Wood-Ljungdahl pathway and were characterized as homoacetogenic bacteria producing acetic acid under minimal medium conditions utilizing hydrogen and carbon dioxide as substrates. RNA stable isotope probing showed that homoacetogenesis is the principal hydrogen sink in the foregut of macropods (1). It is generally accepted that the substantial presence of autotrophic acetogens is one of the reasons that macropods have low-to-no methane production when performing pre-gastric fermentation of feed material (13, 14). Ruminants, on the other hand, rely on methanogenesis from methanogenic archaea for their hydrogen consumption, producing large quantities of methane gas as the final product (6). The hydrogen utilization by these microbes creates a syntrophic relation of methanogens to the fermentative microbes in the rumen. In essence, this relationship directly determines the redox potential by favoring the oxidation of intracellular co-factors that are essential for continuing the glycosidic pathway without the slow regeneration of NADH by NADH-ferredoxin oxidoreductase caused by hydrogen accumulation, as seen in Fig. 1 (15–17). Therefore, this relationship, known as interspecies hydrogen transfer, is present in a broad range of herbivores that rely on fermentative fiber digestions (2, 6, 16, 18). In general, fermentative bacteria are known to have a versatile metabolism and are able to consume many different substrates, allowing them to thrive in competitive environments such as the gastrointestinal tract of animals. Homoacetogens are not an exception, as many known homoacetogens can metabolize carbon sources such as monosaccharides. This allows these microbes to have a metabolism, which is far more diversified than the role of methanogenic archaea in the rumen (19). The fermentative breakdown of complex biomass such as lignocellulosic materials occurs through different well-known steps as shown in Fig. 1, where the final fermentation products are hydrogen and carbon dioxide produced by the acidogenic and acetogenic bacteria. While hydrogen and carbon dioxide are converted to methane or acetic acid, VFAs are converted by acetogens or taken up by the animal. Methanogens are typically the most common hydrogen sink in the foregut of ruminants, with macropods being the largest grazing animals known to rely on an alternative route through homoacetogenesis (4, 18, 20, 21).

Anaerobic fermentation processes have also been increasingly important for the bioeconomy (22, 23). Homoacetogenesis has gained momentum as a viable alternative for the production of renewable acetic acid to substitute acetic acid produced from fossil fuels (24). There is an increased interest in developing techniques for inhibiting methanogenesis in ruminants to curb methane production from livestock activities (25). Furthermore, the opportunity for eliminating methane production for producing VFA from the fermentative breakdown of complex organic materials, the so-called arrested anaerobic digestion, is gaining increased interest (26, 27). When hydrogen consumption in the fermentative process is inhibited, the system loses efficiency for hydrolytic breakdown and fermentation of the input materials due to the thermodynamic imbalance of the overall process. Therefore, it is important to substitute hydrogen consumption with other microbes utilizing hydrogen such as homoacetogenic bacteria (4, 26). The bacterial population and archaeal populations have been observed to change as ruminants age, with the colonization of acetogenic bacteria appearing in young ruminants followed by increased methanogen activity over time (28, 29). Acetic acid molar ratios also change as the animal ages, increasing in the first weeks and leveling off after a few weeks (30, 31).

Due to the role of homoacetogens in macropods, it is necessary to examine the potential of these microbes to substitute hydrogenotrophic methanogens in ruminants. The present study focuses on the identification and cultivation of homoacetogens bacteria from fecal samples of red kangaroo (*Macropus rufus*) and wallabies (*Macropus rufogriseus*). A stable mixed homoacetogenic culture from a joey kangaroo is further characterized, and the microbial community responsible for acetic acid production is identified (4, 32, 33).

## MATERIALS AND METHODS

A collection of kangaroo fecal samples was obtained from Kangaroo Ranch in Fall City, WA, USA. The samples were transported in an ice bath while stored under anaerobic conditions. Fecal samples were collected from adult red kangaroos (*Macropus rufus*) and Bennet wallabies (*Macropus rufogriseus*). The feces were collected directly after being dropped at a major grazing area that consisted of 10 individual adults. The joey kangaroo sample was collected from an individual 2-month-old nursing joey red kangaroo.

### Culture development from adult macropods and a joey red kangaroo

Homoacetogenic culture development was done by performing dilutional batch fermentation in 120-mL serum vials with 50-mL active volume under 10 psi pressure and 200 rpm of agitation in an orbital shaker at 37°C. The initial batch was inoculated with 5% (wt/vol) kangaroo and joey kangaroo fecal samples in 120-mL serum vials with 50 mL of liquid basic anaerobic (BA) medium (34). The minimum media culture was supplemented with 0.1% yeast extract (YE), along with gaseous substrate addition by pressurizing the headspace of serum vials at 10 psi with a gas mixture of hydrogen-carbon dioxide 70%:30% or hydrogen/nitrogen 70%:30% (Oxarc, Pasco, WA, USA).

Each condition was tested in duplicate and the vials were incubated for 14 days with and without the addition of 10-mM bromoethanosulfonate (BES) to test the impact of inhibiting potential methanogenesis. Besides hydrogen-carbon dioxide we further tested two other substrates glucose (5 g/L) and corn stover at 10% wt/vol with a gas phase of nitrogen/carbon dioxide of 70%:30%. The production of acetic acid, gas production, and gas composition were measured at the start of the experiments and after 7 and 14 days for all the vials. These initial batch tests were followed by four subsequent serial dilution batch tests utilizing 10% (vol/vol) of the previous batch as inoculum. After the fourth serial fermentation, the cultures of the highest dilution were used for further testing. The cultures from the fifth fermentation cycle are presented (see Fig. 4) with the pressure readings taken after 14 days with hydrogen-carbon dioxide as substrate. The naming of the cultures refers to the origin of the culture tested. After 7 days, all cultures were repressurized to 10 psi.

### Characterization of the joey kangaroo consortia

Hungate tubes were used for serial dilution to prepare a highly enriched joey kangaroo inoculum. After stabilization, the highly enriched culture grew with a constant generation time and growth rate in BA medium (34) supplemented with 0.1% YE. This culture was used for our further experiments with the joey kangaroo culture.

### Growth rate of the joey kangaroo stable culture

Three 120-mL serum vials with 50 mL of BA media, 0.1% YE, and a headspace of 30%:70% carbon-dioxide/hydrogen gas at 10 psi were inoculated in a laminar flow hood (Labconco,USA) with 10% vol/vol stable joey kangaroo culture and incubated at 37°C for 55 h at 200 rpm in a shaker incubator MaxQ 400 (Fisher Scientific, USA). Optical density (OD) measurements were performed every 2 h to determine the doubling times using a spectrophotometer at 600 nm and correlated with the average dry weight (DW) measured obtained from drying 1 mL of the homoacetogenic culture in three crucibles overnight at 70°C. The linear correlation of OD and DW was used to determine biomass (*X*). The specific growth rate and doubling times were determined at the beginning of the exponential phase through equations 1 and 2, respectively, where *x*_*i*_ and *x*_*f*_ denote biomass in gram per liter at the initial and end of exponential growth, respectively, and *t*_*i*_ and *t*_*f*_ refer to initial and final time points, respectively, of the exponential growth phase (35):


(1) $$\mu =(ln(x_{f}/x_{i}))/(t_{f}-t_{i})$$

(2) td=(ln2)/μ

### Carbon balance of the joey kangaroo stable culture

A duplicate 2-L glass bottle with 1-L sterilized BA media with 0.1% YE was inoculated with 10% vol/vol of joey kangaroo culture during exponential growth and pressurized to 7 psi with a 70%:30% hydrogen-carbon dioxide gas mixture. These cultures were fermented for 72 h at 37°C with an agitation of 120 rpm in a shaker incubator ISF-7100 (Lab Companion, USA) for determining the mass balance during the fermentation. Headspace gas pressure was measured through a rubber tube attached to the rubber stoppers with a bourdon-tube pressure gauge capable of measuring vacuum and adapted with a syringe Luer lock on its socket. Gas pressure was measured every 2 h of fermentation until −15 psi was measured, where it was determined as a complete gaseous substrate consumption.

Ideal gas law (equation 3) was used to quantify carbon dioxide in the headspace of the 2-L flask:


(3) n=PV/RT

where *n* is the number of moles of gas; *P* is the atm pressure of headspace; *V* is the volume in the headspace; *T* is for temperature in Kelvin; and *R* is the gas constant.


(4) $$4H_{2}+2CO_{2}\to C_{2}O_{2}H_{4}+2H_{2}O$$

Based on the stoichiometry equation (equation 4), the carbon conversion efficiency (CCE) was calculated by dividing the carbon molecular weight of acetic acid by the total weight of the carbon dioxide summed with the amount of carbon from YE consumed assuming 37% carbon weight composition as shown in equation equation 5. The values for calculated carbon weight and CCE are shown in Table 1.


(5) $$CCE=C_{2}O_{2}H_{4}{\;}_{\left(CW\;produced\right)}/(\;CO_{2}{\;}_{\left(CW\;consumed\right)}+{YE}_{\left(CW\;consumed\right)})$$

**TABLE 1:** Carbon balance of 1-L batch fermentation of the joey kangaroo culture

Component	Input (g-C)	Ouput (g-C)	CCE	
CO_2_	0.251 ± 0.03	0.030 ± 0.05	70.25% ±3.4%	Substrate
C_2_O_2_H_4_	0.248 ± 0.10	0.499 ± 0.19		Product
Biomass^*a*^	0.146 ± 0.01	0.199 ± 0.04		
Yeast extract^*b*^	0.37	0.287^*c*^ ±0.13		
Total carbon	1.015 ± 0.14	1.015 ± 0.15		

^*a*^ The carbon content of biomass was calculated using the standard elemental biomass formula (CH_1.8_O_0.5_N_0.2_).

^*b*^ Carbon content of yeast extract was assumed to be 37%.

^*c*^ Calculated by the difference of total carbon input – measured carbon output.

### Analytical analysis

Acetic acid concentrations were measured using high-performance liquid chromatography (HPLC). Analysis was done using an UltiMate 3000 HPLC system (Dionex, Sunnyvale, CA, USA) with an Aminex 87H Column 250 4.6 mm (Bio-Rad Laboratories, Hercules, CA, USA) and a Shodex RI-101 refractive index detector. Sulfuric acid (4 mM) in water was used as the eluent, flowing through the 87H column at a constant flow rate of 0.6 mL/min in a constant temperature oven at 60°C. The total time for analysis of the fermentation sample was 25 min. HPLC analyses were conducted by collecting 1 mL of the liquid samples (fermentation effluent) from each batch into 2-mL Eppendorf tubes. The samples were centrifuged at 14,000 rpm for 10 min, and 100 µL of the supernatant analyzed was diluted in 900-µL MilliQ water for HPLC analysis. The gas composition of headspace was performed by collecting a 10-mL sample from the headspace of vials using a syringe with a Luer-lock valve and injecting it in the Universal Gas Analyzer, UGA Series (Stanford Research Systems, Sunnyvale, CA, USA). Biomass concentrations were monitored by sampling 1 mL of culture using a Luer-lock syringe with needles and measuring the absorbance at 600 nm using a Genesys 20 spectrophotometer (Thermo Scientific, USA).

### DNA isolation

Gram staining of the joey kangaroo culture used for the mass balance revealed that a significant number of bacteria were Gram positive. DNA extraction protocol for Gram-positive bacteria was thus followed. Isolation of DNA from the juvenile kangaroo was done by centrifuging 10 mL of culture sample at 8,000 rpm for 10 min and freeze-drying the cell pellet overnight. The next day, frozen pellet was treated with a bead beater, and 750 µL of cetyltrimethylammonium bromide (CTAB) buffer (2% CTAB, 100-mM Tris 8, 20-mM EDTA, and 1.4-M NaCl) was added. Vortexed samples were incubated at 65°C for 1 h; 300-µL phenol:chloroform was added; and the sample was then centrifuged at 14,000 rpm for 5 min.

The supernatant was then transferred to a new 1.5-mL Eppendorf tube, and the phenol:chloroform extraction was repeated. The final supernatant was then dissolved in an equal amount of 2-propanol and centrifuged at 8,000 rpm for 10 min. The supernatant was discarded, and the precipitate was air-dried. The concentration and purity of the DNA were tested using the NanoDrop 1000 spectrophotometer (NanoDrop Technologies,Wilmington, DE, USA) and Qubit 4 Fluorometer (Thermo Fisher Scientific). The isolated DNA purity and quantity can be shown as measured by NanoDrop and Qubit. The ratios of DNA:impurities and DNA:RNA, as well as their concentrations, above 20 ng/µL, were in the acceptable range. DNA electrophoresis of these two juvenile kangaroo cultures further confirmed successful genomic DNA extraction with a similar band size of >1,000 bp, consistent with genomic DNA.

### Genomic DNA sequencing and microbial community analysis

The extracted DNA was normalized to 20 ng/µL, and the sequencing libraries were prepared using a MetaVX 16S rDNA Library Preparation Kit (Genewiz), which targets the V3 and V4 hypervariable regions in the 16S rRNA gene of bacteria. Sequencing was conducted using a 2 × 250 paired-end configuration with an Illumina MiSeq instrument (Illumina, San Diego, CA, USA) according to the manufacturer’s instructions. The base calls were made by internal Illumina software. Sequencing data were converted to FASTQ format with Illumina’s bcl2fastq v.2.17 software. The forward and reverse sequencing data files were generated.

To ensure a robust analysis of the microbial community, we employed two distinct computational pipelines to analyze the raw 16S rDNA sequences. This approach allows for comparison of the results with both historical and recent data. Through these pipelines, we identified the dominant operational taxonomic units (OTUs) as well as the amplicon sequence variants (ASVs) present within the samples:

1. Primer sequences were removed using “cutadapt.” The trimmed sequences were imported into QIIME v.1.9.1 (36). The paired-end reads were joined using “vsearch.” The sequences with <200 bp or with a mean quality score of ≤20 were filtered using “deblur.” The chimeric sequences were identified and removed using “uchime.” The remaining sequences were clustered into OTUs against the SILVA v.119 database pre-clustered at 97% sequence identity.
2. The raw sequences were imported into QIIME v.2 2023.5 (37).

The forward and reverse sequences were denoised, clustered, and chimera-filtered using “DADA2” configured for paired-end demultiplexed data (38) with a truncation length of 250 bp. The classifier Silva 138–99 was used to classify the clustered 16S rRNA sequences and to generate a taxa table (39–41). The QIIME v.2 artifacts were imported into R v.4.3.2 using the “qiime2r” package (42) and converted into a phyloseq object. Richness, Shannon index, Simpson index, Fisher diversity, and abundance-based coverage estimator (ACE) were calculated using the phyloseq object in R. Both OTUs and ASVs were normalized over the total amount of reads in each case to calculate the relative abundances at each taxonomic level.

### Quantitative PCR

Quantitative PCR was performed to quantitatively compare *acsB* genomic sequences to our model homoacetogenic strain of *Acetobacterium woodii* after cultivation for 7 days in BA media with 0.1 YE used previously for quantification and identification of homoacetogenic activity in *A. woodii* (43, 44). Quantitative PCR (qPCR) of isolated DNA was done following the procedure described in references (42, 45). The following were added to the Eppendorf tubes: 10 µL of SsoAdvanced Universal SYBR Green Supermix, 2 µL of forward primer for a final concentration of 500 nM, 2 µL of reverse primer for a final concentration of 500 nM, and template or genomic DNA for a final concentration of 100 ng. The total reaction volume for each sample was 20 µL. The reaction tubes were sealed with an optically transparent lid, vortexed thoroughly, spun down briefly to remove air bubbles, and placed in the MJ MiniOpticon (Bio-Rad Laboratories) real-time PCR instrument. The acetyl-coenzyme A synthase (ACS) gene forward and reverse primers AF1 5′-CTYTGYCAGTCMTTYGCBCC-3′ and AR1 5′-CCCATAAABCCYGGDGTYTG-3′ were selected based on Gagen et al. (44, 46). The target positions in *Moorella thermoacetica acsA*/*acsB* for the forward and reverse primers were 3836–3855 and 4232–4251, respectively, designed to amplify 416 bp of the *acsB* gene. The PCR thermal cycling consisted of one cycle for polymerase activation and DNA denaturation at 95°C for 7 min and 40 cycles for denaturation at 95°C for 15 seconds, followed by annealing/extension and plate read at 60°C for 1 min.

## RESULTS AND DISCUSSION

### Homoacetogenic culture developments

In the first series of batch fermentations inoculated with the fecal materials from adult and joey kangaroos, there was acetate production for all three different substrates: corn stover, glucose, and hydrogen-carbon dioxide in all samples. In the rumen, the prevalence of the methanogenic archaeal population has been identified to be closely associated with protozoa and other hydrolytic microorganisms that produce carbon dioxide and hydrogen besides volatile fatty acids, as they degrade the complex polymers.

The glucose and corn stover were used as substrates to observe if the same hydrolytic activity was present and necessary for using polymeric lignocellulosic materials in the form of corn stover (7, 47, 48). From the production of carbon dioxide and hydrogen using both glucose and lignocellulosic biomass, we found that hydrolytic activities were present in the fecal material (49, 50). The activity of homoacetogens was also observed by consuming carbon dioxide and hydrogen produced during fermentation. All the batches that had an acetate production of over 1 g/L with and without BES were used as inoculum for further fermentations except for the cultures cultivated under glucose, which over time produced lactic acid as the main product. Two vials from the samples from adults with glucose as substrate without BES further produced 1.29 *±* 0.19 mM of methane after 7 days of fermentation. These samples were then eliminated from further studies.

**Fig 2:**
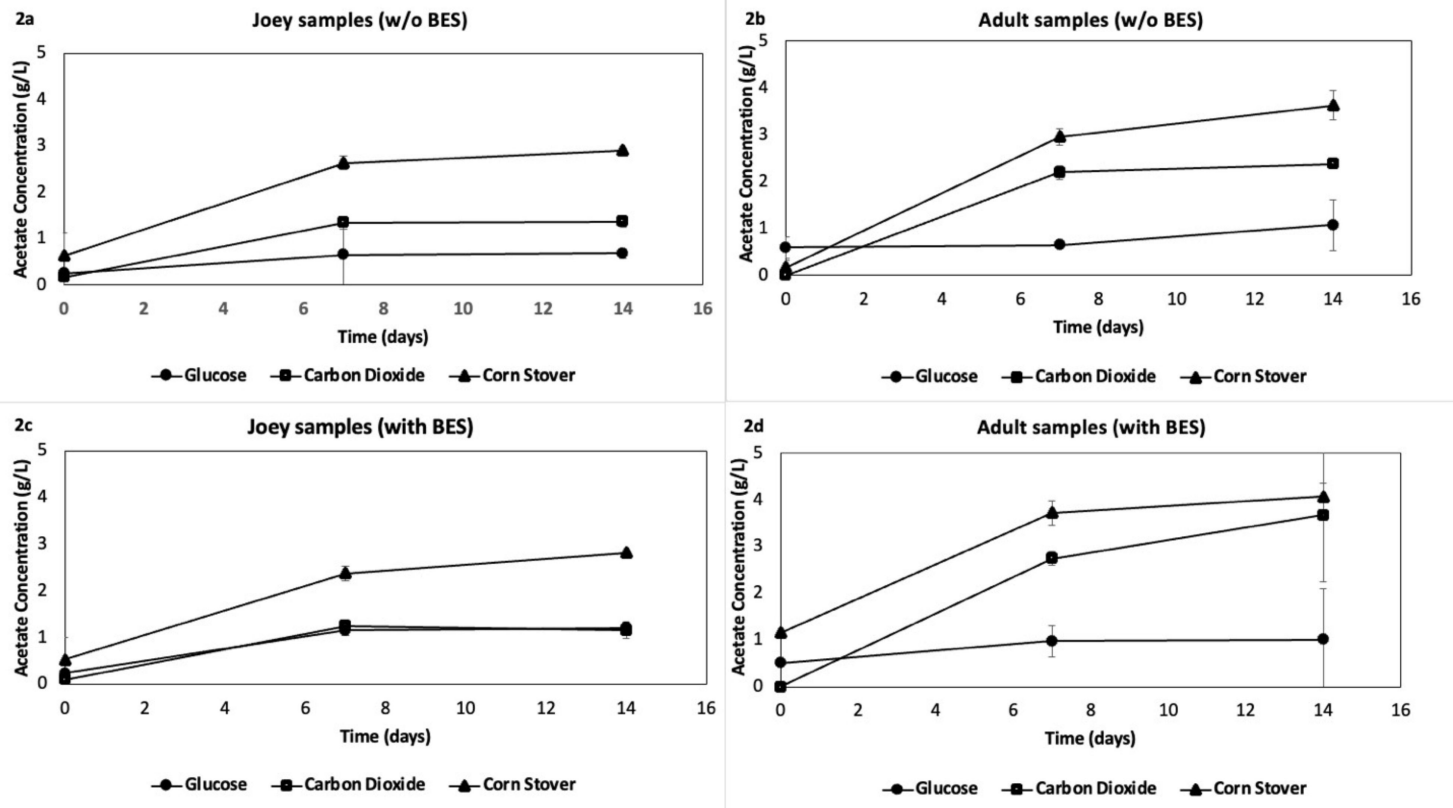
Cultures with fecal inoculum from adult kangaroos (b and d) and joey kangaroos (a and c) using three different carbon sources.

**Fig 3:**
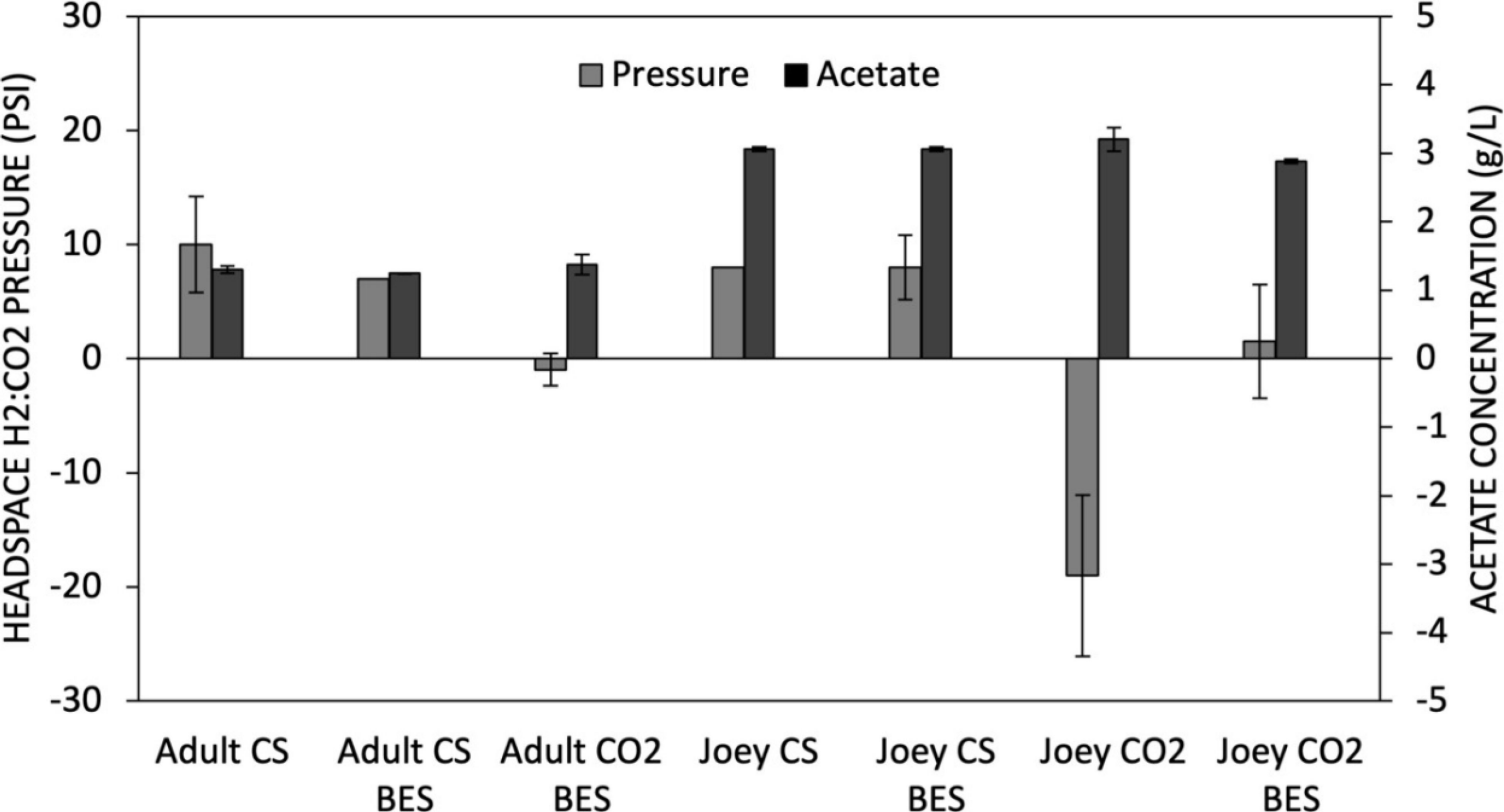
Stable cultures (fifth cycle) of adult and joey kangaroo samples grown for 14 days with hydrogen-carbon dioxide.

More acetic acid was found in the adult kangaroo samples (Fig. 2b and d) of up to 4 g/L compared to the joey kangaroo samples (Fig. 2a and c), where the highest concentration was 3 g/L. BES did not present any significant hindrance to acetic acid production, as shown in Fig. 2. Adult and joey kangaroo samples cultivated with corn stover and joey kangaroo samples grown with hydrogen-carbon dioxide were selected for further studies due to their higher acetate production. Consumption of hydrogen and carbon dioxide was also observed, though lower, indicating the possible presence of homoacetogens in the corn-stover cultures. The consecutive fermentations (four cycles) showed similar results as the first cultivation. We theorized that the joey kangaroo samples would have a more active microflora of homoacetogens, as this has been observed in young suckling herbivores, including cattle and macropods (4). The samples inoculated with the joey kangaroo culture and hydrogen-carbon dioxide as substrate showed a vacuum as a result of gas conversion into acetic acid, with the concentration of acetate at 1.72 *±* 0.28 g/L at 7 fermentation days (data not shown). At 14 days, all cultures with hydrogen-carbon dioxide as substrate showed a pressure below 5 psi, with the exception of the cultures from the corn-stover batch series, which showed no lowering, and maintained their headspace pressure as shown in Fig. 3.

**Fig 4:**
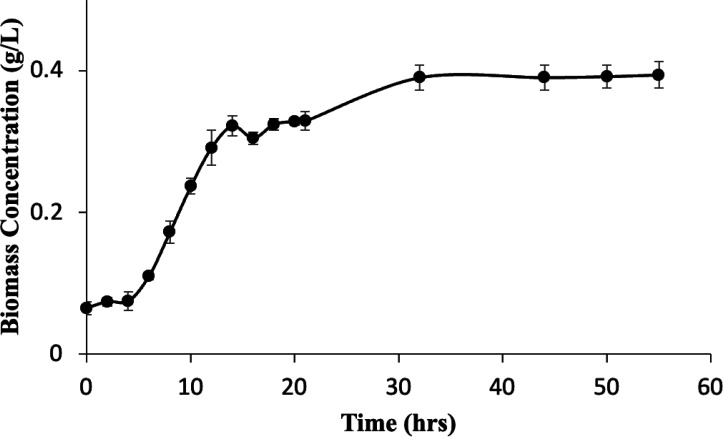
Growth curve of joey kangaroo consortia in 50 mL. BA media with 0.1% YE in serum vials and 10% inoculum from joey kangaroo culture with 10 psi H_2_:CO_2_ 70%/30% gas phase. Measurements were performed in triplicate with error bars representing standard error.

The joey kangaroo culture grown on hydrogen-carbon dioxide had the highest reduction in pressure compared to the samples from adult kangaroos and was selected for further characterization of the homoacetogenic populations. The increased acetate production in the joey kangaroo cultures cultivated without BES compared to cultures with BES may be due to the broad inhibition of microorganisms by BES as previously described. The cultures producing high concentrations of acetic acid from a corn-stover serial fermentation showed that, when switched to a hydrogen-carbon dioxide substrate, they were not able to fully consume the substrate, as vacuum was not observed in any “CS” culture in Fig. 4. The observance of vacuum only in cultures cultivated with carbon dioxide as sole carbon source indicates that the acetogenic population preferably uses organic substrate when multiple carbon sources are available. There was no methane produced in any of the samples presented in Fig. 4. The higher consumption of hydrogen-carbon dioxide in cultures previously grown on hydrogen-carbon dioxide as substrate is indicative of a selection during repeated transfers toward autotrophic organisms, and those cultures were therefore selected for our study.

### Growth kinetics

The specific growth rate of bacteria under hydrogen-carbon dioxide is informative for understanding the capability of a microorganism or a consortium to serve as a hydrogen sink in anaerobic systems. The growth curve of the stable joey kangaroo culture is presented in Fig. 4.

As shown in Fig. 4, the joey kangaroo culture was entering exponential growth after 6 h. The doubling time of the joey kangaroo culture was 3.54 ± 0.53 h, with a growth rate of 0.199 ± 0.032/h. In comparison, the average doubling time of hydrogenotrophic methanogens with hydrogen-carbon dioxide is from several hours to over a day at 37°C (50–52), showing that the joey kangaroo culture could be competitive with methanogens with sufficient hydrogen availability (25, 53).

Concentrations of products formed were taken at initial time *T* (4) and at 14-h *T* (14). By calculating the difference between g-C output and input in Table 1, we can determine that 0.251 g-C was converted to acetic acid formation. Besides acetic acid, there were no other products detected by HPLC; therefore, no other carbon products other than biomass were assumed to be formed in relevant quantities. With that assumption, the YE played a secondary role as carbon source, as more than two times the amount of carbon from CO_2_ was consumed for product and biomass formation compared to YE.

### Taxonomic composition

**Fig 5:**
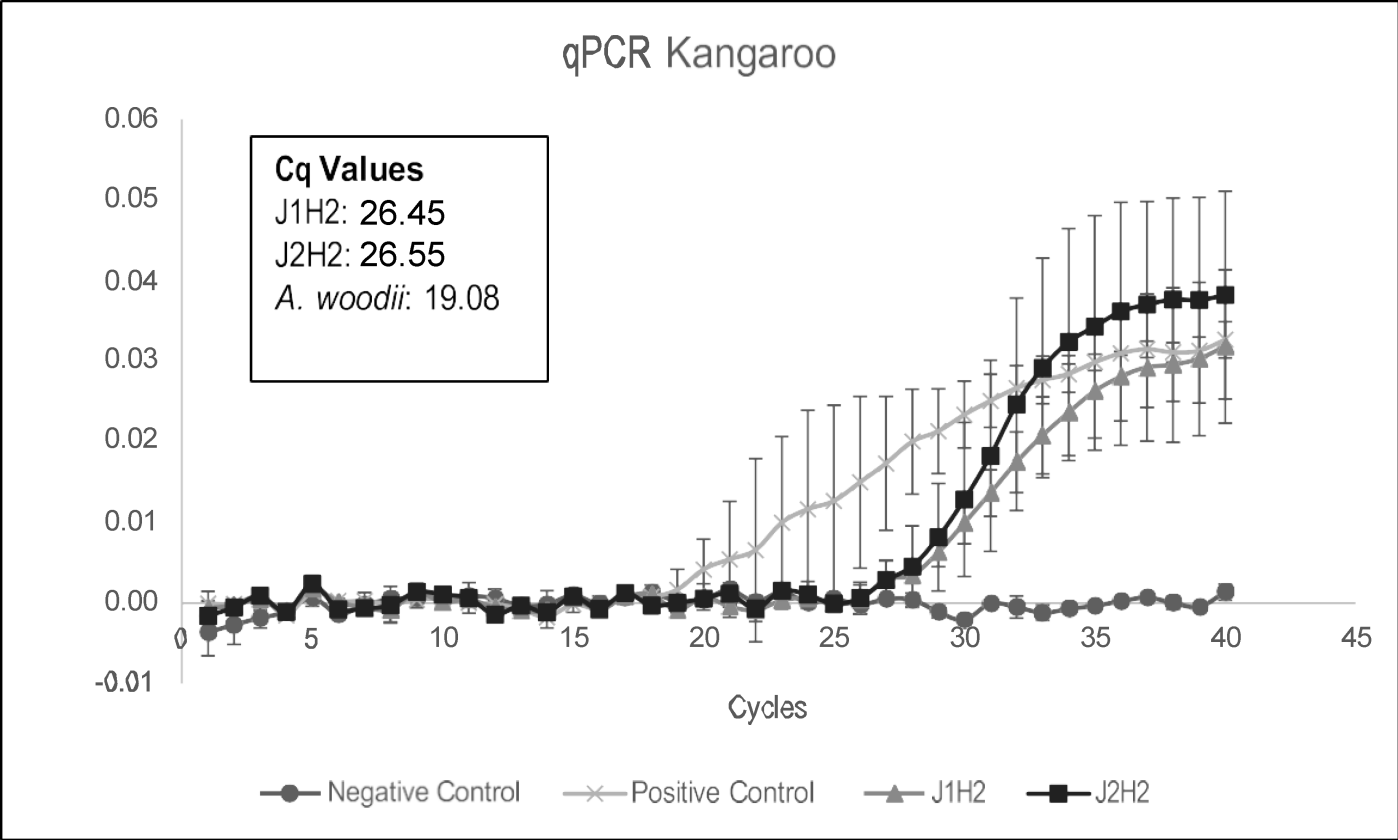
The *acsB* comparative quantification of the two joey cultures. *Acetobacterium woodii* was used as positive control for the ACS gene and no template control as a negative control.

To ensure that the culture did indeed convert the hydrogen-carbon dioxide through the Wood-Ljungdahl pathway as done by homoacetogens, we tested the culture for the presence of the ACS gene using qPCR analysis. ACS is a crucial enzyme in the Wood-Ljungdahl (WDL) pathway that is highly active during reductive acetogenesis (54). Both cultures from the joey kangaroo samples showed a high amount of ACS gene copies (CQ value below 29 cycles) as shown in Fig. 5, indicating that a significant population of bacteria in both cultures were homoacetogens (46, 55).

**Fig 6:**
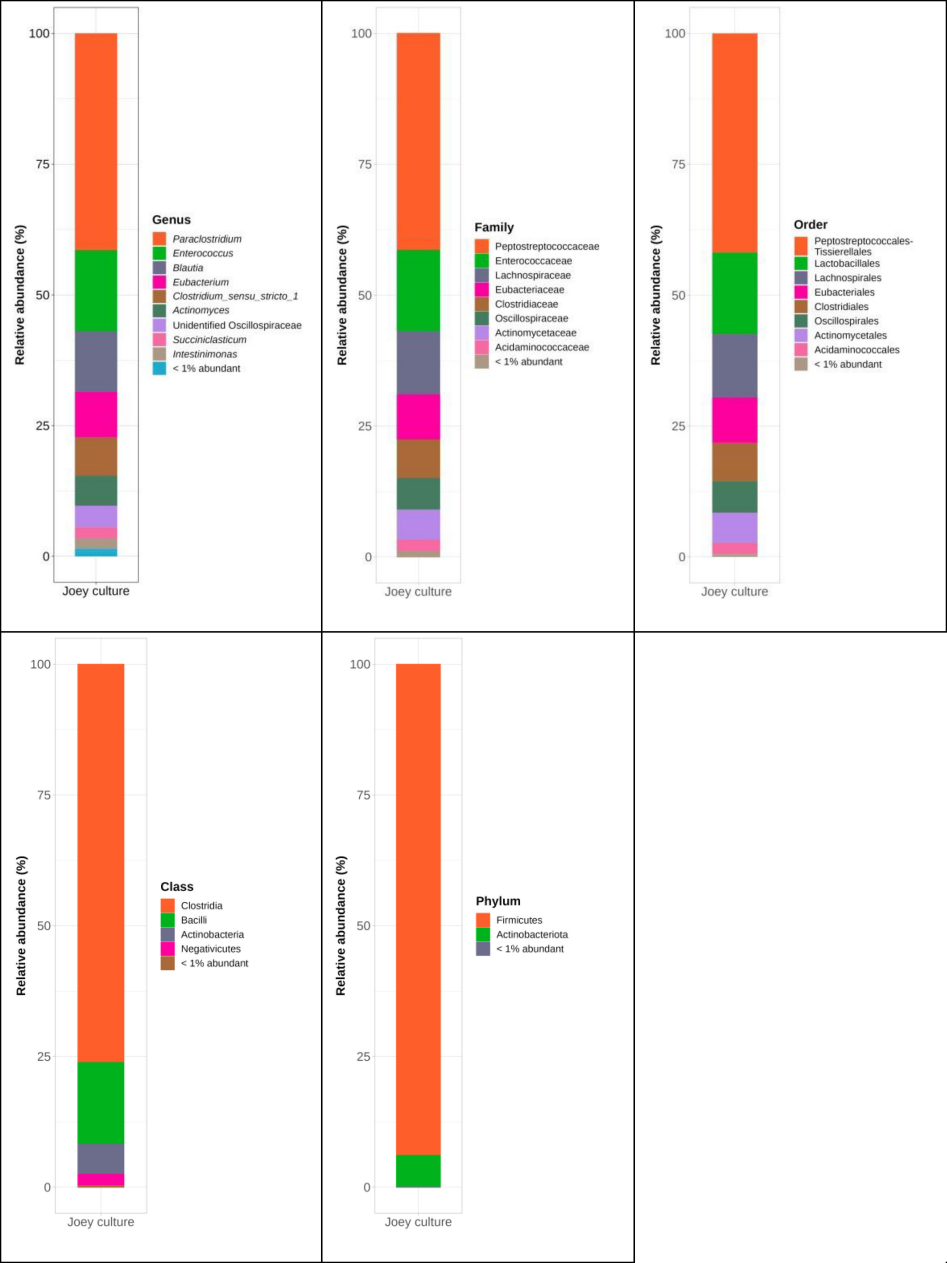
Taxonomic composition of joey kangaroo consortia against SILVA138.

The ACS gene copies were quantified using qPCR to determine the relative abundance of homoacetogenic bacteria in both cultures cultivated with joey kangaroo fecal samples. The results of qPCR are shown in Fig. 5. ACS gene amplicons were detected in both DNA isolates from the joey kangaroo and *A. woodii*, while the no-template control (NTC) showed no dimers being formed, as expected. Figure 5 clearly shows that homoacetogenic bacteria are active in the joey kangaroo cultures. Sequencing of the joey kangaroo cultures showed several unclassified species (86.7% of the species was unknown in the most recent database). However, the rest of the species found in the sample correspond well to previous sequencing studies of the foregut and feces samples from macropods, where over 70% of the gene copies belonged to the genus of Clostridia (1, 56, 57). Other prevalent genera of our joey kangaroo samples were Bacilli and Actinobacteria, from the *Actinomyces* genus as seen in Fig. 6. Nonparametric estimators were used to determine the diversity of the bacterial community with the evolutionary distance of 0.03 (or 97% 16S rRNA gene sequence similarity). The ACE indicator was 33 ± 2.4, consisting mainly of three different bacterial classes (Clostridia, Bacilli, and Actinobacteria). In contrast to what has previously been found in studies of samples from the foregut of kangaroos, our studies did not identify Bacteroidetes (1, 56). This group has generally been found to play an important role in the digestive process of kangaroos, being mostly involved in the degradation of complex carbohydrates (58). Its absence indicates a selection toward homoacetogenic bacteria during our cultivation of the joey kangaroo samples.

As shown in Fig. 6, The *Paraclostridium* genus was the most abundant, corresponding to almost half of the reads in the joey kangaroo cultures, followed by *Enterococcus* and *Blautia*. Against SILVA 119, two species were successfully identified as *Clostridium bifermentans* and *Blautia producta*. *C. bifermentans* represented 48.5% of the species found during sequencing. *C. bifermentans* (reclassified as *Paraclostridium bifermentans*) has been identified in human feces and in manure but has neither previously been identified from the foregut nor from fecal samples of macropods. It is further not classified as a homoacetogenic bacterium (59). However, the presence of the WDL pathway has been characterized in the Clostridia Cluster XI, including *C. bifermentans* (60, 61). Though not specified against SILVA 138–99, *Paraclostridium* spp. were the most abundant genus in the culture, with over 40% abundance. Another identified genus identified in both against both databases was *Blautia* accounting for 12% percent of the population. This bacteria genus has well-known homoacetogens such as *Blautia producta* and is commonly found in the rumen and other anaerobic environments (62). In fact, all of the genera identified in these consortia are Firmicutes bacteria, with the exception of *Actinomyces*. The presence of *Actinomyces* spp. was surprising as very few studies have previously identified this genus as part of the GI tract of macropods (1, 13, 63), and none, to our knowledge, have been classified as homoacetogenic. Still, species of this genus have been described to have genes for enzymes present in the Wood-Ljungdahl pathway, which indicates the presence of ACS activities (64). Bacteroidetes, as a highly abundant phylum found in the rumen and also identified in the GI tract of kangaroos, is notably absent in this consortium. This phylum has been associated with hygenotrophic activity, mainly propiogenesis, which relies on lactic acid production from carbohydrates, a substrate absent in our cultures. Their absence, therefore, could be related due to lack of available carbon sources in the medium of our *in vitro* cultivation.

### Conclusion

Homoacetogens were successfully cultivated from fecal samples of macropods, as demonstrated through multiple repeated transfers of cultures producing acetate as the sole product when grown on hydrogen-carbon dioxide. Homoacetogens from a juvenile kangaroo were found to produce the highest amount of acetic acid compared to cultures from adult wallabies and red kangaroos. Growth of the joey kangaroo culture on hydrogen-carbon dioxide demonstrated a doubling time of 3.5 ± 0.2 h, faster than previously described for the homoacetogenic model organism *Clostridium ljungdahlii* (65). This is the first study, to our knowledge, where *Actinomyces* bacteria have been identified from macropods’ fecal samples and sustained their presence throughout a series of batch fermentations utilizing a hydrogen-carbon dioxide headspace substrate mixture. We theorize that they may have a competitive advantage under the specific conditions to thrive either by their capability to metabolize the gaseous substrate or through syntrophic interactions with the homoacetogenic populations present. The results of this study show that fecal samples from macropods are a rich source for the bioprospection of homoacetogens capable of thriving under anaerobic conditions, with the aim of producing acetic acid without the influence of methanogens. This study highlights the diversity of the homoacetogenic population in macropods, offering insights into the role of homoacetogenesis in these animals. Besides, our finding offers potential for reducing the methane production from ruminants by direct bioaugmentation of ruminants with cultures from macropods and, in particular, from joey kangaroos.

## Data Availability

Data are fully available upon request to the corresponding author.
